# Application of Sigma metrics in assessing the clinical performance of verified *versus* non-verified reagents for routine biochemical analytes

**DOI:** 10.11613/BM.2018.020709

**Published:** 2018-06-15

**Authors:** Shuang Cao, Xiaosong Qin

**Affiliations:** Department of Medical Laboratory, Shengjing Hospital of China Medical University, Shenyang, China

**Keywords:** quality assessment, Sigma metrics, method decision chart, total allowable error

## Abstract

**Introduction:**

Sigma metrics analysis is considered an objective method to evaluate the performance of a new measurement system. This study was designed to assess the analytical performance of verified *versus* non-verified reagents for routine biochemical analytes in terms of Sigma metrics.

**Materials and methods:**

The coefficient of variation (CV) was calculated according to the mean and standard deviation (SD) derived from the internal quality control for 20 consecutive days. The data were measured on an Architect c16000 analyser with reagents from four manufacturers. Commercial reference materials were used to estimate the bias. Total allowable error (TEa) was based on the CLIA 1988 guidelines. Sigma metrics were calculated in terms of CV, percent bias and TEa. Normalized method decisions charts were built by plotting the normalized bias (bias_a_: bias%/TEa) on the Y-axis and the normalized imprecision (CV_a_: mean CV%/TEa) on the X-axis.

**Results:**

The reagents were compared between different manufacturers in terms of the Sigma metrics for relevant analytes. Abbott and Leadman’s verified reagents provided better Sigma metrics for the alanine aminotransferase assay than non-verified reagents (Mindray and Zybio). All reagents performed well for the aspartate aminotransferase and uric acid assays with a sigma of 5 or higher. Abbott achieved the best performance for the urea assay, evidenced by the sigma of 2.83 higher than all reagents, which were below 1-sigma.

**Conclusion:**

Sigma metrics analysis system is helpful for clarifying the performance of candidate non-verified reagents in clinical laboratory. Our study suggests that the quality of non-verified reagents should be assessed strictly.

## Introduction

Quality control (QC) is the essence and core of a laboratory, assuring the quality of results that are critical for clinical diagnosis and patient care. To produce reliable results, the quality of testing is usually monitored with Levey-Jennings charts to provide a good review of analytical precision in particular, but establishing the internal quality control (IQC) program for analytical phase is not sufficient (*1*). To complement it, external quality assurance (EQA) programs were established. However, it is still difficult to provide a direct and integrated assessment of the performance of the analysis system. In recent years, a summary analytical assessment method based on Sigma metrics is widely accepted. It was originally proposed for industry, and has been applied to any process, especially the analytical process in clinical laboratories (*2*).

Sigma metrics combine bias, imprecision, and total allowable error (TEa) and make them become an objective and integrated assessment of the quality of analytical tests. Sigma metrics analysis provides a standardized scale for comparing the quality of testing results by evaluating the performance of testing procedure or methods (*3*-*5*). In addition, laboratories can also choose the appropriate QC rules based on the review of QC results on the Sigma scale (*6*, *7*).

In clinical laboratories, the entire testing system consists of instruments, reaction reagents, calibrators and control materials. When the reagents used on the instruments come from the same manufacturer, they are routinely designated as the original verified reagents, which are usually recommended by reagent manufacturers. There are other alternate verified reagents, also known as permitted reagents, which have been verified for using on the instruments from different manufacturers. The non-verified reagents are referred to as the reagents used on the instruments from different manufacturers but have not been verified. In China, although the international recognized instruments for routine biochemical analytes are widely used, like Architect, Beckman and Hitachi, considering lower cost and other factors, many clinical laboratories, especially the laboratories in underdeveloped areas, usually prefer to choose the permitted and non-verified reagents for biochemical analytes instead of original testing kit. However, this is not recommended by the instrument companies.

For a new non-verified reagent system, laboratory professionals must perform a series of tests to investigate if bias or imprecision of the obtained results might have a clinical impact on patients. The requirement for the acceptability of experimental reagent can be expressed as total allowable error, a combination of random error and systematic error, which are expressed by CV and bias, respectively. However, though some guidelines like Clinical and Laboratory Standards Institute (CLSI) or International Organization for Standardization (ISO) 15189 are well accepted in many laboratories, no global consensus currently exists on which allowable total error to choose for assessing acceptability. Sigma is a metric that measures the performance of a process quantitatively. In statistics, six sigma represents an ideal situation with the possibility of producing defects in a process of only 3.4 defects per million (*8*). Based on the Sigma metrics, the acceptability of the test systems can be compared in a standardized scale. Therefore, for the laboratory professionals, Sigma metrics perhaps is the more appropriate method to assess the acceptability of a non-verified reagent system.

The analytical Sigma metrics has been used to compare the performance of methods, instruments and analytes, but few data are available for reagents, particularly the data comparing the verified and non-verified reagents for biochemical analytes.

In this study, we calculated the Sigma metrics for clinical chemistry analytes tested with the reagents from four manufacturers, including verified and non-verified reagents on an Architect c16000 chemistry analyzer (Abbott Diagnostics, Abbott Park, USA) to compare their performance on Sigma scale. Our goal was to provide a practical and instructional approach in assessing the performance of verified and non-verified reagents. This is of great significance to the quality control of clinical laboratories in China.

## Materials and methods

### Study design

We conducted a prospective study in the clinical laboratory in Shengjing Hospital, using an Architect c16000 chemistry analyser. The study was conducted over the period of one month from July 2017 to August 2017. Reagents for biochemical analytes were provided by four manufacturers, including Abbott (Abbott Park, USA), Leadman (Beijing, China), Mindray (Shenzhen, China) and Zybio (Chongqing, China). The reagents of Abbott and Leadman had been verified on Abbott Architect c16000. They were regarded as verified reagents. The reagents of Mindray and Zybio had not been verified on Abbott instruments. They were non-verified reagents. All tests were performed on the same instrument using the same reagent probe to ensure the comparability of results. The same lots of reagents were used during the study period. As the mandatory and representative analytes for enzyme activity and metabolism, alanine aminotransferase (ALT, without pyridoxal-5-phosphate), aspartate aminotransferase (AST, without pyridoxal-5-phosphate), urea and uric acid (UA) were tested with the reagents from manufacturers of Abbott (ALT: Lot 48963UQ02; AST: Lot 36351UN16; urea: Lot 82424UN16; UA: Lot 40999UN16), Leadman (ALT: Lot 703161C; AST: Lot 703232C; urea: Lot 704072C; UA: Lot 704133D) and Mindray (ALT: Lot 14011701; AST: Lot 14021701; urea: Lot 199300334; UA: Lot14121701) except Zybio (ALT: Lot 170501; AST: Lot 161201; urea: Lot 170501), simultaneously to acquire the internal quality control (IQC) data and external quality control (EQC) data. No Zybio reagent was available for UA assay.

### Methods

The internal QC data were analysed against the recommended calibration intervals in the reference manual. For reagents of Abbott, ALT and AST assays were calibrated with water. Urea and UA assays were calibrated with Multiconstituent Calibrator (Lot 78377; Abbott Diagnostics, Abbott Park, USA). For the reagents of Leadman, multi-biochemical calibrator (Lot 611021G; Leadman, Beijing, China) was used for calibration. ANDOX CAL2351 (Lot 914UE; Randox Laboratories Ltd, United Kingdom) was used for calibration of the reagents of Mindray and Zybio.

Internal QC materials, Lyphochek Assayed Chemistry Control (Lot, 14490; Bio-Rad Laboratories, Irvine, USA) (two levels, normal and pathological values) were tested once daily, using reagents from four manufacturers concurrently. The coefficients of variation (CV) were calculated with the mean values of internal QC data during 20 consecutive days.

Bias data were acquired from the commercial reference materials with target values, provided by National Institute of Metrology (Beijing, China) for UA (GBW09157) and Medical device laboratory (Beijing, China) for urea (GBW(E)090547), ALT and AST (GBW(E)090593). The relative bias was calculated with the average results of five repeated tests and the target values.

According to the Clinical Laboratory Improvement Amendments of 1988 (CLIA 1988), the recommended allowable error (TEa) values for analytes are presented in Table 1 (*9*).

**Table 1:** Recommended allowable total error values

**Test**	**ALT**	**AST**	**urea**	**UA**
**TEa (%)**	20	20	9	17
TEa - allowable total error according to the recommendation from the Clinical Laboratory Improvement Amendments of 1988 (CLIA 1988) (*9*). ALT – alanine aminotransferase. AST – aspartate aminotransferase. UA – uric acid.				

### Statistical analysis

Statistical data, including mean, standard deviation (SD), and CV were analysed with SPSS version 21.0 (SPSS Inc., Chicago, USA) (*10*). The CV was calculated as CV_IQC_(%) = (standard deviation × 100) / laboratory mean_(IQC)_. Percent bias was calculated as Bias(%) = (target value of reference material - mean result of our laboratory) × 100 / (target value of reference material). Sigma metrics were calculated from CV, percent bias and TEa for the analytes using the formula Sigma metrics = (TEa - Bias) / CV_IQC_ (*11*). The mean and standard deviation for laboratory parameters were presented with as many decimals as the results are usually reported on the laboratory test report (*12*). The calculated parameters, such as CV, bias and Sigma metrics, were presented with three decimals.

A normalized method decision chart was drawn up plotting the normalized bias (bias_a_ = bias / TEa) on the Y-axis and the normalized imprecision (CV_a_ = mean calculated CV / TEa) on the X-axis, considering the different TEa (*13*). Different zones displayed in the graph correspond to the different Sigma metrics levels. The closer a method’s plotted point is to the origin, the better its Sigma metrics, and more reliable the results provided by this method are (*6*).

## Results

Table 2 provides the daily QC data for four analytes tested with reagents from different manufacturers. When comparing the means of calculated CV, we found that Abbott reagents gave lower CVs for AST, urea and UA than the reagents of other manufacturers, especially Leadman and Zybio, but slightly poorer than the Mindray reagent for the ALT assay (2.70% *vs* 2.63%).

**Table 2:** Internal quality control data for the analytes tested

**Analytes**	**Reagent manufacturers**	Mean*	**SD**	**CV** **(%)**	**Mean** **CV (%)^†^**
**ALT** **(U/L)**	Abbott	28	0.93	3.37	2.70
		97	1.96	2.03	
	Leadman	28	1.04	3.67	3.25
		94	2.68	2.83	
	Mindray	29	0.86	3.02	2.63
		97	2.18	2.24	
	Zybio	33	1.63	4.98	3.84
		101	2.71	2.69	
**AST** **(U/L)**	Abbott	42	0.85	2.01	1.66
		189	2.47	1.31	
	Leadman	41	1.13	2.74	2.24
		190	3.28	1.73	
	Mindray	44	0.96	2.19	1.82
		199	2.88	1.44	
	Zybio	44	1.61	3.61	2.40
		195	2.33	1.19	
**urea** **(mmol/L)**	Abbott	5.5	0.12	2.21	2.41
		17.1	0.44	2.6	
	Leadman	5.6	0.26	4.63	4.63
		15.6	0.72	4.62	
	Mindray	6.0	0.14	2.37	3.02
		16.5	0.06	3.67	
	Zybio	5.9	0.41	7.01	5.18
		16.7	0.56	3.34	
**UA** **(μmol/L)**	Abbott	286	2.98	1.04	0.82
		586	3.53	0.6	
	Leadman	289	2.73	0.95	0.87
		572	4.45	0.78	
	Mindray	289	4.33	1.50	1.14
		577	4.45	0.77	
SD – standard deviation. CV – coefficient of variation. ALT – alanine aminotransferase. AST – aspartate aminotransferase. UA – uric acid. *The mean data of internal quality control with two levels were accumulated for 20 consecutive days. ^†^The mean CV were the average data of 2 QC levels of CV.					

Table 3 shows the bias calculated by the average values of inaccuracy data, using commercial reference materials. The percent bias with Leadman reagents was 0.22% for ALT, 0.85% for AST and 0.03% for UA assay, much better than the reagents of other manufacturers. As for urea assay, Abbott reagents (2.19%) provided optimal bias, followed by Leadman (6.65%), Zybio (8.01%) and Mindray (8.32%).

**Table 3:** Bias for the analytes tested

**Analytes**	**Reagent manufacturer**	Target value*	**Mean_ref_^†^**	**Bias (%)**
**ALT** **(U/L)**	Abbott	72	75	4.06
	Leadman		72	0.22
	Mindray		75	4.64
	Zybio		81	12.50
**AST** **(U/L)**	Abbott	157	165	5.01
	Leadman		156	0.85
	Mindray		153	2.73
	Zybio		167	6.11
**urea** **(mmol/L)**	Abbott	4.6	4.6	2.19
	Leadman		4.3	6.65
	Mindray		5.0	8.32
	Zybio		4.9	8.01
**UA** **(μmol/L)**	Abbott	341	338	0.97
	Leadman		341	0.03
	Mindray		340	0.26
ALT – alanine aminotransferase. AST – aspartate aminotransferase. UA – uric acid. *The given value of reference material (RM). ^†^The average of results, which were measured with the commercial reference material five times.				

The Sigma metrics values calculated for each control level and calculated from the mean calculated CV are shown in Table 4 in terms of reagent manufacturers. The reagents of Leadman achieved the highest Sigma metric value for ALT (6.09), followed by Abbott (5.90), Mindray (5.84) and Zybio (1.96). All reagents performed well for the AST and UA assays, as evidenced by Sigma metrics higher than 5 (for AST: 9.03 with Abbott, 8.57 with Leadman, 9.52 with Mindray and 5.79 with Zybio; for UA: 19.55 with Abbott, 19.62 with Leadman and 14.75 with Mindray). For the urea assay, the Abbott reagent achieved the best performance, reaching 2-sigma and approaching 3-sigma (2.83). The results of other reagents were below 1-sigma (0.51, 0.23 and 0.19, respectively).

**Table 4:** Calculated Sigma metrics for the analytes tested

**Analytes**	**Reagent manufacturer**	**Calculated Sigma** metrics*	**Calculated** **Sigma metrics_mean_^†^**
**ALT**	Abbott	4.73	5.90
		7.85	
	Leadman	5.38	6.09
		6.98	
	Mindray	5.09	5.84
		6.86	
	Zybio	1.51	1.96
		2.79	
**AST**	Abbott	7.47	9.03
		11.45	
	Leadman	7.00	8.57
		11.09	
	Mindray	7.88	9.52
		11.96	
	Zybio	3.84	5.79
		11.63	
**urea**	Abbott	3.08	2.83
		2.62	
	Leadman	0.51	0.51
		0.51	
	Mindray	0.29	0.23
		0.19	
	Zybio	0.14	0.19
		0.3	
**UA**	Abbott	15.41	19.55
		26.72	
	Leadman	17.86	19.62
		21.76	
	Mindray	11.18	14.75
		21.68	
ALT – alanine aminotransferase. AST – aspartate aminotransferase. UA – uric acid. *The Sigma-metric was calculated with the CV of each QC level, respectively. ^†^The Sigma-metric was calculated with the mean of CV, which is available in Table 2.			

**Figure 1:**
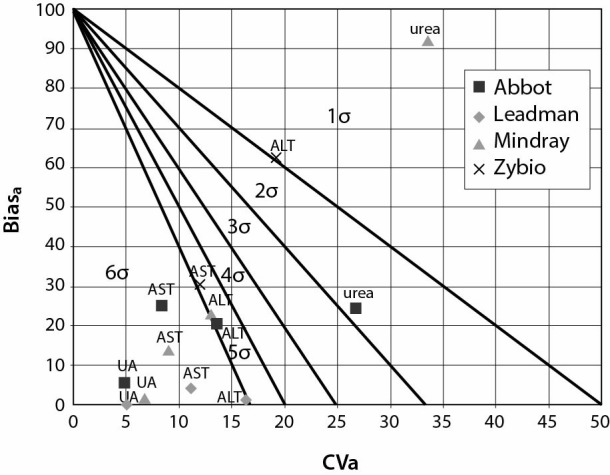
Normalized method decision chart for the analytes tested. Y-axis represents the normalized bias as bias_a_; X-axis represents the normalized imprecision as CV_a_. Diagonal lines separate the graph into different sigma zones, which correspond to the different Sigma metrics levels.

To provide a quick visual decision about the acceptability of verified and non-verified reagents for biochemical analytes, a normalized method decision chart was drawn for different reagents (Figure 1). The chart shows assay performance by the reagents of Abbott, Leadman and Mindray are mostly located in the regions of 5-sigma or 6-sigma. The Zybio’s plotted points fall in region 5-sigma only for the AST assay, and are located in unacceptable zones for the ALT and urea assays. However, the plotted points drawn for Leadman and Zybio for the urea assay were not shown in Figure 1, because their calculated CV_a_ exceeded the maximum scale on the X axis.

## Discussion

Sigma metrics is a standard management model for improving laboratory quality by identifying and minimizing errors. It is applicable for assessing and directly comparing the quality of analytical processes. The performance and equality are consistent with Sigma metric value (*14*). The ideal analytical system should have a Sigma metrics of 5 or 6 or higher, which is considered as “excellent” or “world class”, respectively, while 3-sigma indicates minimum of 2 levels per day accepted by routine clinical tests (*15*). The Six Sigma Model has been used as a global quality management system applicable to the benchmarking of several clinical chemistry biomarkers. However, there is still no related report from its clinical application and comparison in China. In this study, we found that Abbott and Leadman’s verified reagents provide the better Sigma metric than non-verified reagents (Mindray and Zybio) in reagent-instrument performance for the ALT and UA assays. There is a gap ranging from 0.04-sigma to 4.80-sigma between the reagents of different manufacturers, particularly between the verified and non-verified reagents. This finding suggests laboratories that reagents are not simple commodities and their analytical quality cannot be assumed acceptable and interchangeable.

Abbott reagents provide the best Sigma metric value of 2.83 for the urea assay, but this is still under the basic requirement of 3-sigma. The results of other reagents are below 1-sigma, which is an unacceptable analytical performance. Our results are comparable to the results of Singh *et al.* which were below 2-sigma, but are completely different from Nanda *et al.*’s results with 5-sigma, although total allowable errors (TEa) for calculating the Sigma metrics are taken from the guidelines of CLIA (*3*, *16*). We thought that the difference might be due to the selection of different based-units, as the TEa for urea was defined as two units-based goals in CLIA, 9% or 0.71 mmol/l. The TEa of 9% was chosen in this study as well as Singh *et al.*’s, but the unit of TEa was not specified in Nanda *et al.*’s report. However, given the variability of TEa provided by different guidelines, the Sigma metric vary either. A previous study has also reported that TEa targets from different sources have important impact on the interpretation and application of Sigma metrics in routine laboratory testing (*17*). Briefly, the global consensus for the definition of the TEa is absent and it would be the main barrier to the application of the Sigma metrics analysis.

The analytical imprecision, CV, is the measure of instability of analytical system. In daily laboratory testing, the higher CV means the assay has more variation and less precision. In this study, the CVs were calculated with the daily control data during a period of 20 consecutive days. To ensure assessments’ reliability and stability, the study is preferably conducted over a longer time period. Bias, as a main component that the sigma value is dependent on, estimates the inaccuracy of results and is usually assessed by difference in the means of results like EQA. However, different manufacturers, instruments and methods significantly influence the bias, even the Sigma metric value (*11*). These results suggest that laboratories should be careful to choose the source of the bias and to define the Sigma metrics level of performance required. However, in our study, bias was conducted with true reference materials, making the results traceable and consequential.

There may be such a misunderstanding that high Sigma metrics level means better precision and accuracy. For example, Leadman reagents provide better Sigma metrics values due to higher calculated CV and smallest bias, while Mindray reagents show lower Sigma metrics value due to smaller CV and bigger bias. These data clearly indicate that Sigma metrics is a summary indicator combining multiple key analytical performance parameters.

Burnett *et al.* found that the Sigma metrics values calculated at different concentrations are quite different (*18*). This is also observed in our study. Taking AST for example, the Sigma metrics value of the reagents from four different manufacturers were > 6 at the pathological level (QC2). The performance of these tests at pathological level (QC2) is considered “excellent” and there is no difference in the QC rules that should be implemented. However, the Sigma metric at normal control level (QC1) were also > 6 except Zybio which obtained a Sigma metric of 3.84. This indicates that a combination of QC rules would be used for the reagent of Zybio. Therefore, the clinical performance of reagents may be dependent on concentration of analytes.

One major limitation of this study was that there was no corresponding calibrator available for the reagents of Mindray and Zybio, and a Randox calibrator was used instead. However, Randox calibrator was recommended by the introductions of Zybio. The replaceable calibrator was not declared in the introductions of Mindray, but the assignment of Randox calibrator can be traced back to the internationally recognized reference material or reference method which is consistent with the reagent of Mindray. In spite of this, it maybe still affects the bias and resultant Sigma metrics value for the reagent of Mindray and Zybio.

To conclude, currently many reagents produced in China have emerged and the qualities vary significantly from manufacturer to manufacturer. During reagent selection, it is critical to select with acceptable Sigma metrics level. When clinical laboratories plan to switch reagent systems, our results suggest that the non-verified reagent system generally has relatively lower Sigma metrics level than verified reagent system, but it needs to be noted that this result is not absolute. Besides, the Sigma metrics varies with the reagent, analyte, and concentration. Therefore, Sigma metrics analysis system is helpful for clarifying the performance of candidate non-verified reagents in clinical laboratory. This process should be performed strictly. As the Sigma metrics has been applied not only in the “established” laboratory, but also in developing countries like Ghana and India, *etc* (*16*, *19*). Therefore, our conclusions are instructive not only in China but also in the other developing countries.
